# Dysregulation of YAP by the Hippo pathway is involved in intervertebral disc degeneration, cell contact inhibition, and cell senescence

**DOI:** 10.18632/oncotarget.23299

**Published:** 2017-12-14

**Authors:** Cong Zhang, Feng Wang, Zhiyang Xie, Lu Chen, Arjun Sinkemani, Haomin Yu, Kun Wang, Lu Mao, Xiaotao Wu

**Affiliations:** ^1^ Department of Spine Surgery, Zhongda Hospital, School of Medicine, Southeast University, Nanjing, China

**Keywords:** Hippo pathway, YAP, LATS, intervertebral disc degeneration, nucleus pulposus

## Abstract

The Hippo pathway plays important roles in wound healing, tissue repair and regeneration, and in the treatment of degenerative diseases, by regulating cell proliferation and apoptosis in mammals. Intervertebral disc degeneration (IDD) is one of the major causes of low back pain, a widespread issue associated with a heavy economic burden. However, the mechanism underlying how the Hippo pathway regulates IDD is not well understood. Here, we demonstrate that the Hippo pathway is involved in natural IDD. Activation and dephosphorylation of yes-associated protein (YAP) were observed in younger rat discs, and decreased gradually with age. Surprisingly, Hippo pathway suppression was accompanied by overexpression of YAP, caused by acute disc injury, suggesting a limited ability for self-repair in IDD. We also demonstrated that YAP is inhibited by cell-to-cell contact via the Hippo pathway *in vitro*. Phosphorylation by large tumor suppressor kinases 1/2 (LATS1/2) led to cytoplasmic translocation and inactivation of YAP. YAP dephosphorylation was mainly localized in the nucleus and regulated by the Hippo pathway, whereas YAP dephosphorylation occurred in the cytoplasm and was associated with nucleus pulposus cell (NPC) senescence. Moreover, NPCs were transfected with shYAP and it accelerates the premature senescence of cells by interfered Hippo pathway through YAP. Therefore, our results indicate that the Hippo pathway plays an important role in maintaining the homeostasis of intervertebral discs and controlling NPC proliferation.

## INTRODUCTION

Degeneration and injury of intervertebral discs result in alterations in mechanical and biological behaviors [1]. Intervertebral disc degeneration (IDD) induces neck and low back pain and is one of the most common causes of morbidity. It affects 70–85% of all individuals at some point in their lives and has enormous socioeconomic implications [2]. Treatment options for IDD include: 1) conservative methods using non-steroidal anti-inflammatory drugs (NSAIDs), physical therapy, rest, and lumbar muscle functional exercises; 2) surgical approaches consisting of disc arthroplasty, spinal fusion, and disc replacement; and 3) biomedical engineering techniques to attempt to retard the IDD process, including cell-based therapy, gene transfer, protein injection, and biomaterial implantation [3, 4]. These therapeutic options have proven to be increasingly effective in recent years.

The etiology of IDD is complex, and mechanical, inflammatory, and structural causes can lead to increased nociception [5, 6]. Therefore, future studies aimed at understanding the mechanisms underlying IDD and developing novel therapies are highly warranted.

Yes-associated protein (YAP) is a transcriptional coactivator and negative regulator of the Hippo tumor suppressor pathway. The Hippo pathway is an evolutionarily conserved pathway that regulates diverse cellular processes, such as cell proliferation, differentiation, apoptosis, and tissue size [7]. It has been shown to be involved in the regeneration of several organs, including the heart, liver, nervous system, and intestines, following tissue damage. [8–11] In mammals, the Hippo pathway consists of mammalian sterile 20 like kinases 1/2 (MST1/2) and large tumor suppressor kinases 1/2 (LATS1/2) [12]. Activation of the Hippo pathway results in inactivation of YAP via phosphorylation of ser127 by LATS1/2 [13]. Phosphorylated YAP subsequently translocates and is sequestered to the cytoplasm from the nucleus, via binding to 14-3-3, and is then degraded. In contrast, the Hippo pathway does not trigger YAP phosphorylation, resulting in nuclear accumulation of YAP that drives growth promoting transcription genes, such as connective tissue growth factor (CTGF) and cysteine-rich angiogenic inducer 61 (Cyr61) through the TEA domain (TEAD) transcription factor family.

In this study, we describe the role of the Hippo pathway in regulating IDD in rat tail discs. We hypothesized that the Hippo pathway is involved in the regulation of IDD, the underlying mechanism is related to age and IDD injuries, and the viability and biological features of nucleus pulposus cells (NPCs) are affected differently by the Hippo pathway depending on the cell microenvironment and cell-to-cell contact. A rat disc degeneration model was used, in which rat tail discs were punctured, to characterize degeneration traits and reveal the role of the Hippo pathway compared with natural disc degeneration. Because Hippo signaling was suppressed at 4 weeks after puncture, the observation time was prolonged to 12 weeks postoperatively. Moreover, changes in YAP and YAP phosphorylation were studied to elucidate the regulatory mechanism underlying activation of the Hippo pathway in NPCs.

## RESULTS

### DHI decreased and Pfirrmann grades increased in puncture groups

**Figure 1:**
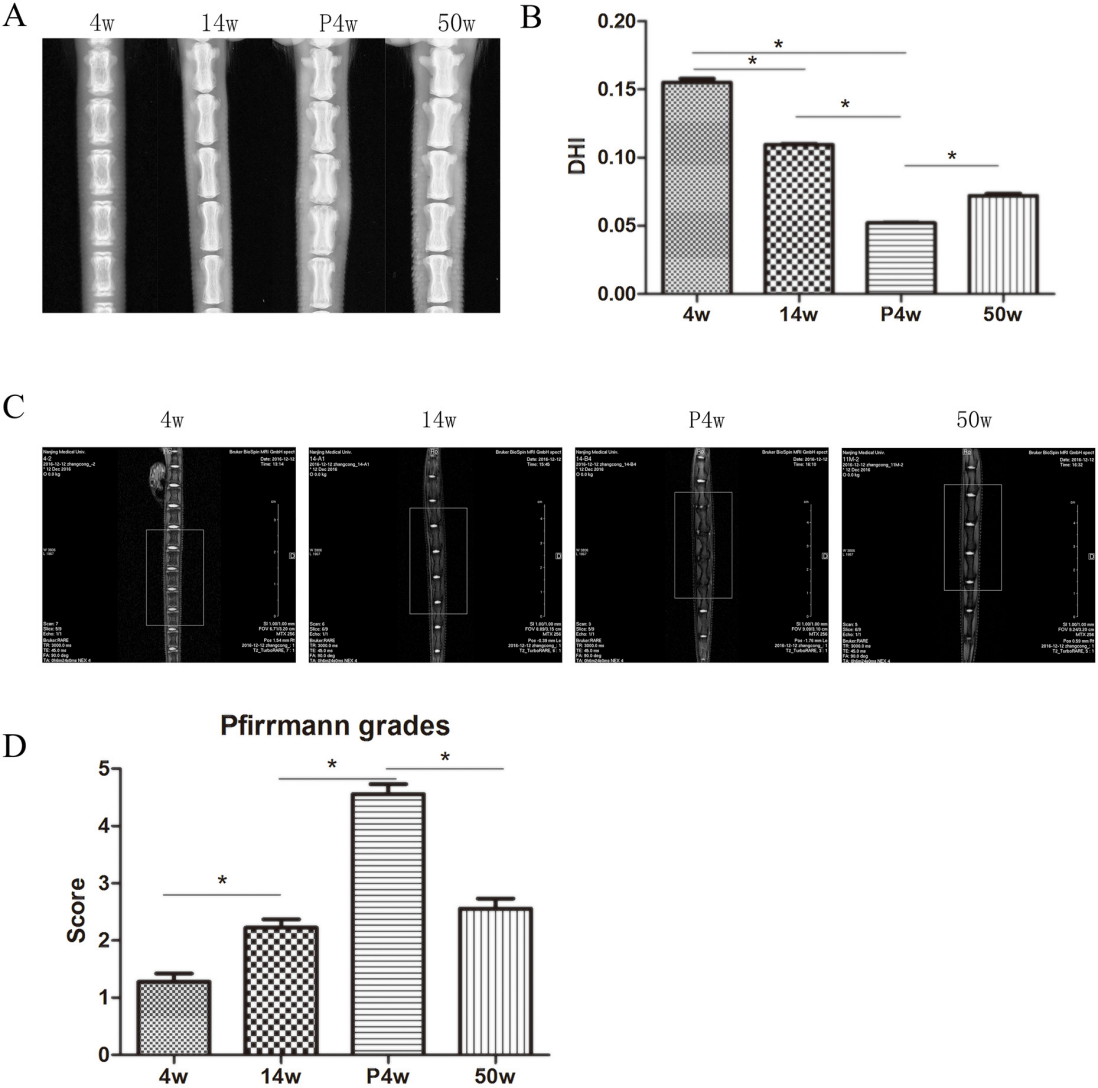
Intervertebral disc degeneration (IDD) occurred gradually in the natural groups **(A)** X-ray assessment of IDD. **(B)** Calculation of disc height index (DHI) from digital radiographs, as described by Han et al. **(C)** IDD imaging was performed using a T2-weighted sagittal magnetic resonance imaging (MRI) system. **(D)** Pfirrmann grades were assigned on the basis of the MRI signals within the nucleus pulposus, endplate changes, and signs of internal disc derangement or tearing (^*^ *P*<0.05).

Changes in DHI in the natural IDD groups are shown in Figure 1A and 1B. The values for the coccygeal 5-6, 6-7, 7-8, and 8-9 discs were averaged for analysis because they had similar features. We observe statistically significant differences in DHI among the natural IDD groups. The IDD values at 14w and 50w were lower than those at 4w, indicating that discs continuously and slowly degenerate with age. Indeed, older discs displayed more serious IDD. Annulus injury induced by needle puncture resulted in significantly decreased disc height, to below that of the non-puncture groups. However, there was no significant decrease in %DHI between the P4w and P12w groups (Figure 4A–4C, Supplementary Figure 1A). These findings demonstrate that a 21 G needle puncture may cause more damage to discs, and that IDD reached a maximum value at 4 weeks after puncture.

**Figure 2:**
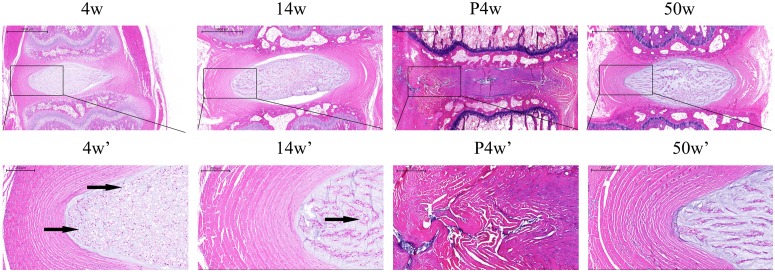
Histological changes in the natural groups and the acute disc injury group, including a magnified view (black arrows: vacuolated notochordal cells)

**Figure 3:**
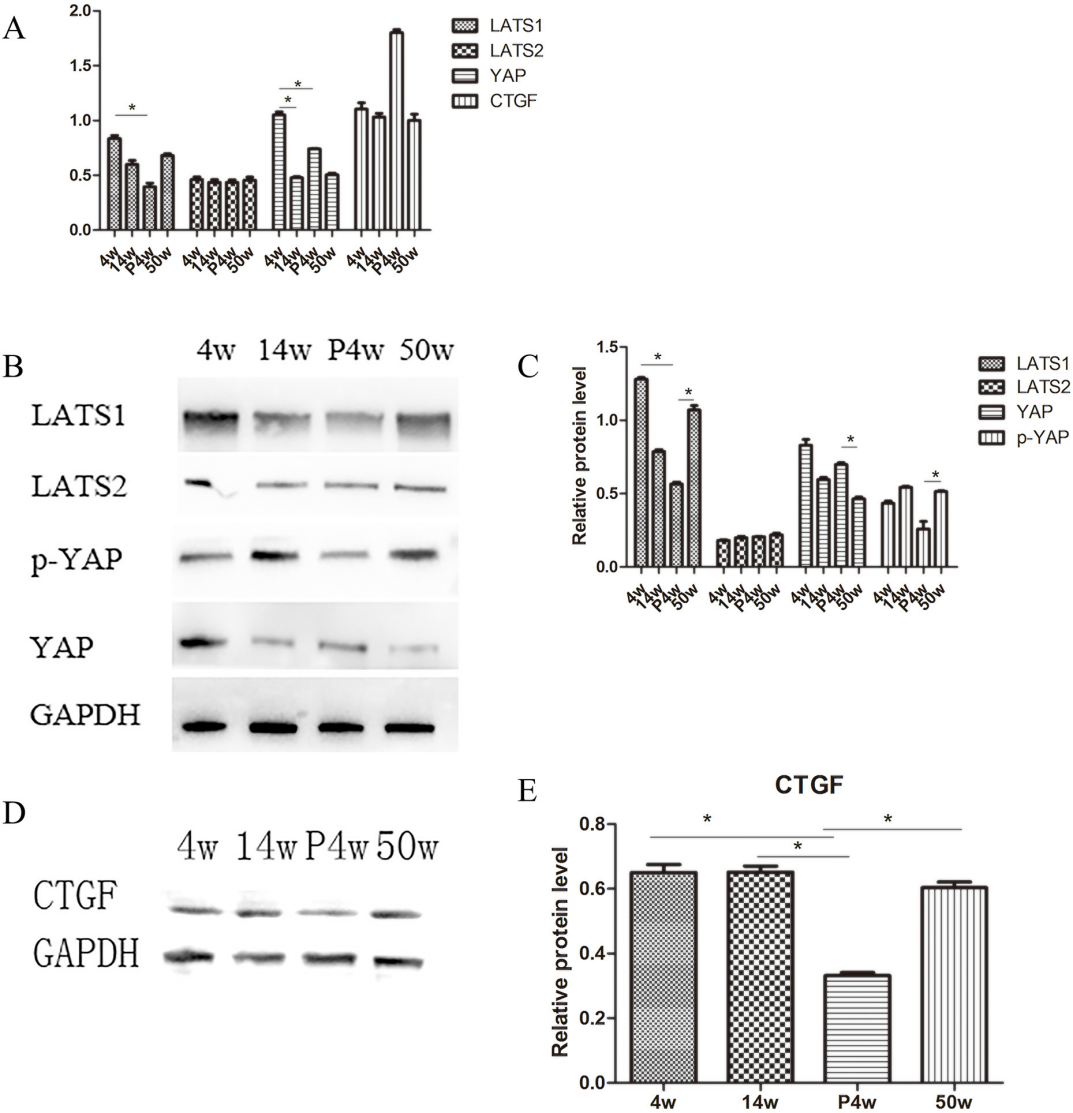
IDD occurred gradually in the natural groups and incomplete compensation of self-repair in acute injury induced IDD **(A)** Quantitative polymerase chain reaction (qPCR) comparing the relative expression of LATS1, LATS2, yes-associated protein (YAP), and connective tissue growth factor (CTGF) in the 4w, 14w, P4w, and 50w groups. **(B–E)** Western blot analysis of Hippo pathway-related proteins using anti-LATS1, anti-LATS2, and anti-YAP (B) and anti-CTGF (D) antibodies. Histogram showing average relative protein levels normalized to glyceraldehyde 3-phosphate dehydrogenase (GAPDH) (C and E) (^*^ *P*<0.05).

**Figure 4:**
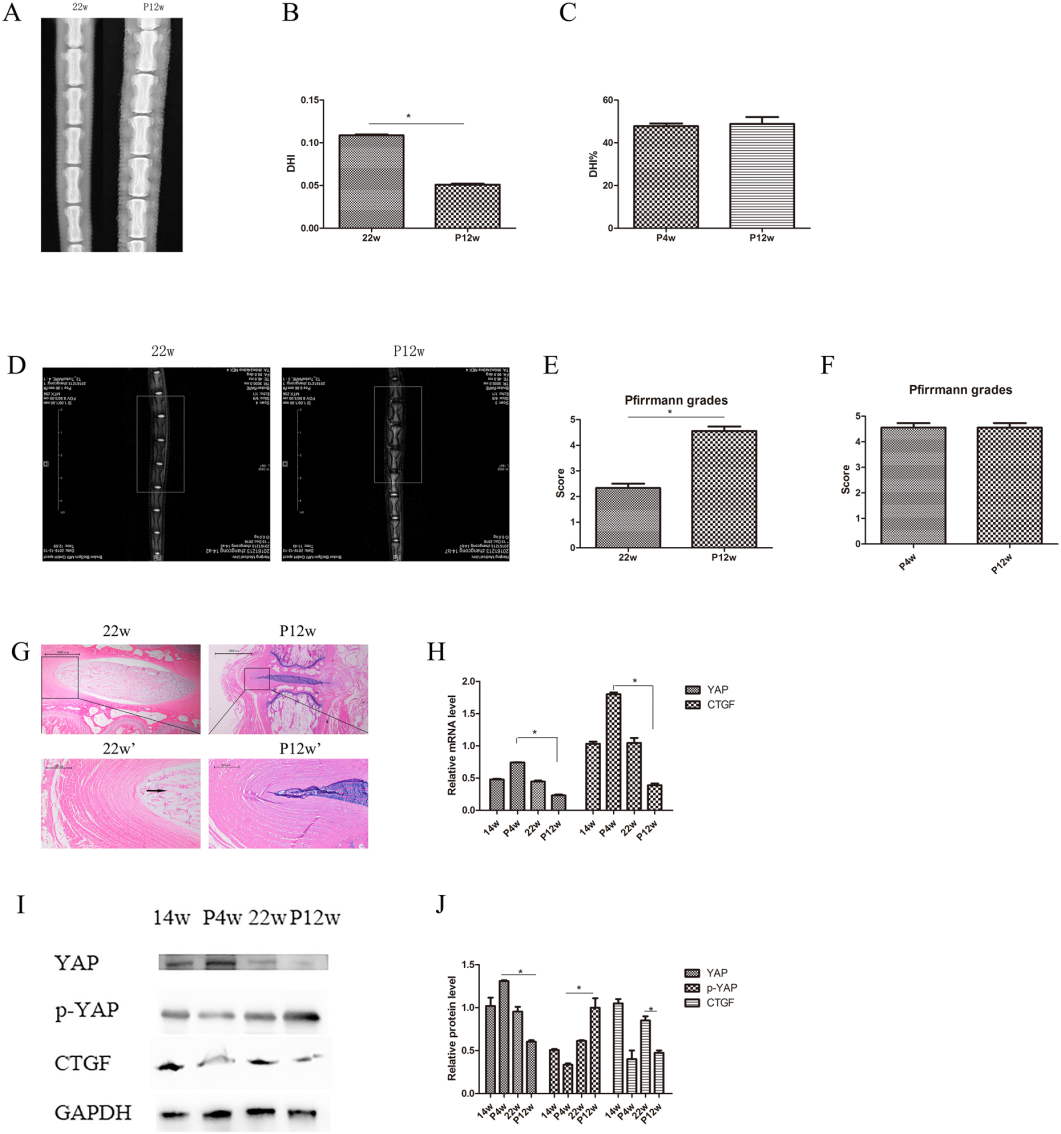
There were no significant differences between the P4w and P12w group on X-ray or MRI examination **(A)** X-ray assessment of IDD in the 22w and P12w groups. (**B** and **C**) Calculation of DHI in the 22w and P12w groups (B) and percentage DHI in the P4w and P12w groups (C) from digital radiographs. **(D)** IDD imaging was performed using a T2-weighted sagittal MRI system. (**E** and **F**) Pfirrmann grades were assigned on the basis of MRI signals. **(G)** Histological changes in the 22w group and the chronic disc injury group (P12w), including a magnified view. **(H)** qPCR comparing the relative expression of YAP and CTGF in the 14w, P4w, 22w, and P12w groups. (**I** and **J**) Western blot analysis of Hippo pathway-related proteins in the 14w, P4w, 22w, and P12w groups using anti-YAP, anti-p-YAP, and anti-CTGF antibodies (I). Histogram showing average relative protein levels normalized to GAPDH (J) (black arrows: vacuolated notochordal cells) (^*^ *P*<0.05).

MRI of natural group tail discs showed normal signals, and the gray scales and Pfirrmann grades increased gradually over time (Figure 1C and 1D). The degenerative disc results of the P4w and P12w groups were similar, showing only dark areas and Pfirrmann grades of 4–5 (Figure 4D–4F, Supplementary Figure 1B). Although significant differences among the natural IDD groups were found, no differences were observed between the 14w and 22w groups, suggesting that the process of IDD is relatively stable in adult rats.

### Histological changes in IDD and incomplete compensation of self-repair in acute IDD injury

To identify IDD patterns, H&E staining was performed to examine the morphology of the NP and annulus fibrosus (AF) (Figure 2 and Figure 4G). Compared with the other groups, the 4w group contained more vacuolated notochordal cells and less small chondrocyte-like cells with even cellular distribution. In the 50w group, the level of vacuolated notochordal cells decreased gradually over time, and NPC clusters and serpentine AF were found, indicating disc degeneration. Compared with the control group, significant disc morphological changes, including small NP areas and an increased number of chondrocyte-like cells, were observed after puncturing. Disrupted borders between AF, NP, and AF serpentine fibers were also found.

**Figure 5:**
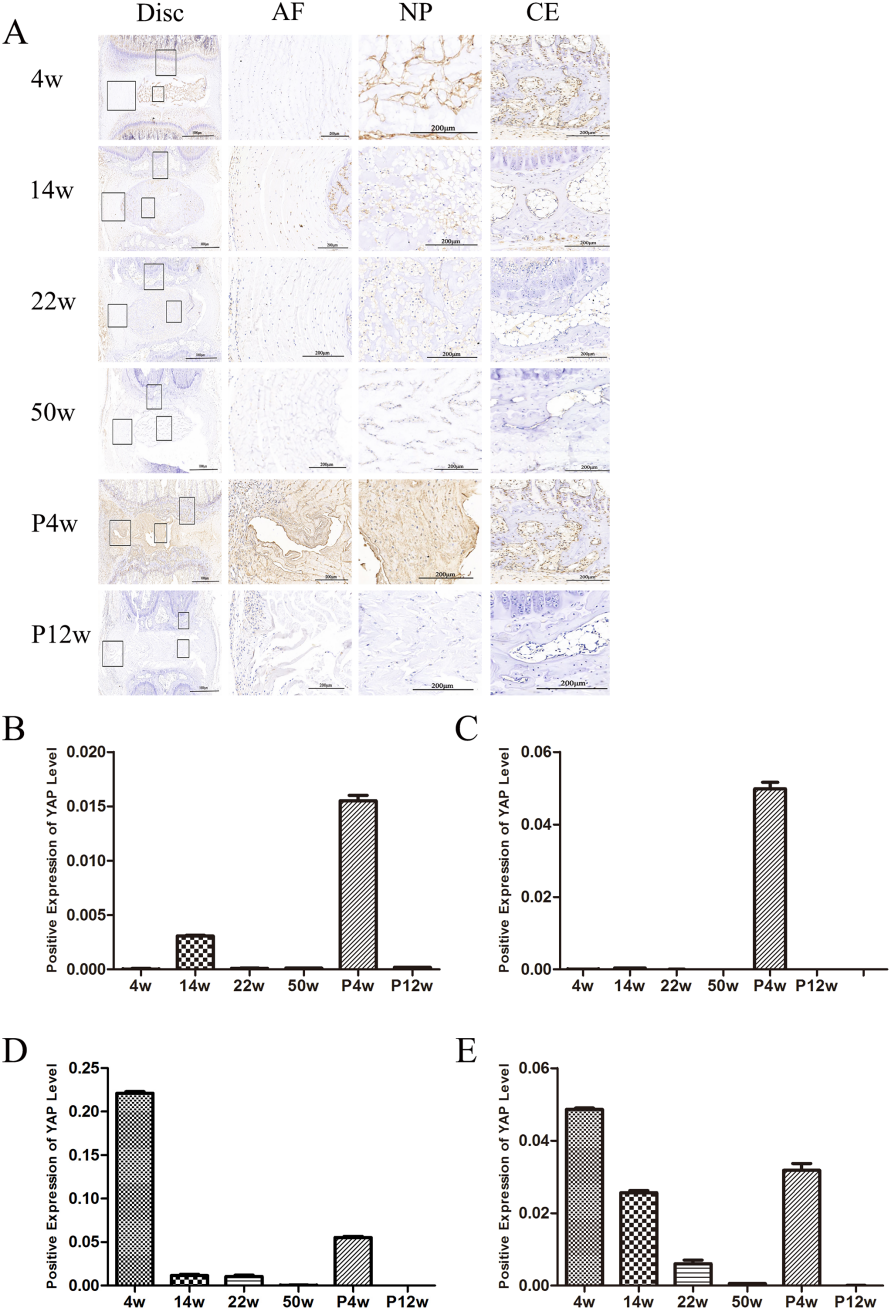
YAP levels decreased in the NP and endplate with age, and Hippo signaling was activated in chronic disc injury-induced IDD **(A)** Immunohistochemical detection of YAP. The right panel shows a magnified view of different sites. **(B–E)** Quantification of YAP levels in the outer annulus, (B) inner annulus, (C) NP, and (D) endplate.

To investigate differences in Hippo signaling between the natural disc degeneration group and the puncture group, P4w rats were studied. Surprisingly, YAP was upregulated in all disc areas (NP, AF, and cartilaginous endplate (CE)), resulting in suppression of Hippo signaling (Figure 5). qPCR and western blot analysis of the NP confirmed these findings. Indeed, the expression of YAP increased, while the expression of phosphorylated (p)-YAP decreased (Figure 3A–3C).

The expression of the genes of interest, YAP and CTGF, in NP tissue was analyzed with qPCR. Younger age was associated with increased expression of both YAP and CTFG proteins and genes. In the P4w group, the gene expression of YAP was consistent with that of CTGF. However, the expression of the CTGF gene was not consistent with the expression of the CTGF protein, considering the self-repair ability of intervertebral discs and incomplete compensation of self-repair (post-transcriptional regulation) (Figure 3D and 3E). To investigate how YAP expression was affected by injury time, the observation time was prolonged to 12 weeks post-puncture, with 22-week old rats selected as controls. In the P12w group, the expression of YAP in all intervertebral disc areas (NP, AF, and CE) was decreased. The gene and protein expression levels of CTGF and YAP were consistent, and both declined in the P12w group (Figure 4H and 4J).

### Hippo pathway suppression in young discs and collagen type 2 levels decreased gradually in the non-puncture groups

To investigate the role of the Hippo pathway in IDD *in vivo*, the coccygeal 5-6, 6-7, 7-8, and 8-9 intervertebral discs of the natural IDD groups were removed and YAP immunohistochemical staining was performed. Additionally, NP tissue was extracted for qPCR and western blot analyses. Interestingly, the expression of YAP at various sites was different, including the outer AF, the inner AF, the NP, and the CE (Figure 5). The expression of YAP in the CE was consistent with that in the NP, and was gradually reduced with increasing age. As for YAP could regulate cell growth and maintain homeostasis, Hippo signaling was suppressed in rats at 4 weeks post-operatively but gradually increased with age, while YAP expression was consistent with natural IDD on MRI, H&E staining, and collagen type 2 staining.

In the 4w group, YAP expression in the inner and outer annulus was low; however, it was increased in the 14w group, with higher expression intensity in the outer annulus. Three major mechanisms could account for this phenomenon. First, at 14 weeks, the disc may have started to degenerate because YAP levels in the NP were lower than in the 4w group. Second, with body growth, the vertebral body and intervertebral disc volume increased, rendering proliferation of the outer AF a good environment for enlarged NP. Third, proliferation of annulus can increase connectivity between the upper and lower vertebral bodies, thereby maintaining mechanical stability of the intervertebral discs. From the examination of NP tissue, YAP expression decreased with age, yet upstream LATS1 levels increased in young rats. LATS1 is regulated at the transcriptional level and is a direct target gene of YAP; LATS1 mRNA levels increase upon YAP activation [14, 15]. Regulation of LATS1 constitutes a negative feedback loop to ensure that overactivation of YAP does not occur and maintain homeostasis of the Hippo signaling pathway. Inconsistent with the trend in YAP expression, the expression of p-YAP decreased gradually with time.

**Figure 6:**
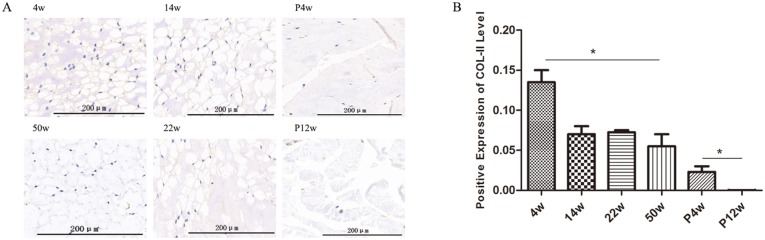
Collagen type 2 levels decreased gradually in the non-puncture groups and significantly decreased in the punctured tail discs **(A** and **B)** Immunohistochemical detection of collagen type 2 (A) and quantification of collagen type 2 present in the NP (B) (black arrows: vacuolated notochordal cells) (^*^ *P*<0.05).

The NP is characterized by a low density of NPCs and a highly abundant extracellular matrix, which contains large amounts of collagen type 2 and proteoglycan [16]. Disc collagen type 2 was quantified using immunohistochemistry, and clear trends were observed in the natural degeneration and puncture groups (Figure 6). In the natural degeneration group, collagen type 2 levels decreased gradually with increasing age as same as H&E staining, unlike in the study by Chen et al.[17], in which collagen type 2 levels increased at 2 weeks post-operatively and then gradually decreased. This difference may be due to differences in observation time after puncturing. In our study, collagen type 2 quantification was not consistent with X-ray and MRI results. Although %DHI and Pfirrmann grades peaked 4 weeks after puncture, collagen type 2 levels were significantly decreased in the P4w group and subsequently gradually decreased in the P12w group (Figure 6A and 6B). These data indicate that tail puncture using a 21 G needle can successfully induce IDD. In addition, the inconsistency of the staining and imaging results between the P4w and P12w groups may be due to imaging being a coarse examination, and thus not capable of detecting changes in the NP. Moreover, the water content loss rate in the NP is slower than internal chemical composition changes.

### YAP responds to cell-cell contact

**Figure 7:**
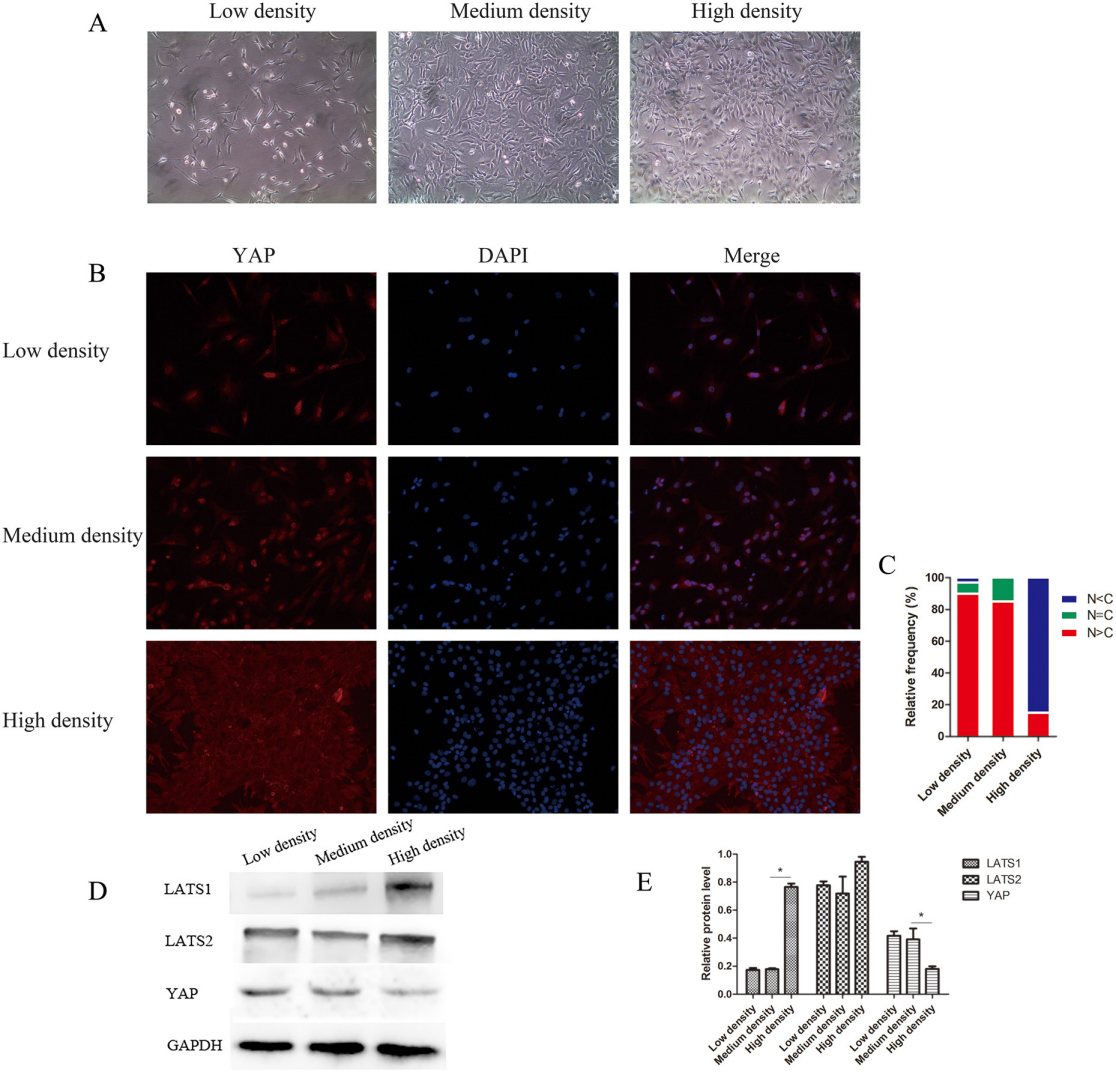
YAP localization and the Hippo pathway are regulated by nucleus pulposus cell (NPC) density **(A)** NPCs were cultured at different densities, ranging from sparse density to confluence. **(B** and **C)** YAP localization is affected by cell density. YAP was stained with anti-YAP antibody (B) and cells in five random views were quantified for YAP subcellular localization (C). **(D** and **E)** Western blot analysis of Hippo pathway-related proteins using anti-LATS1, anti-LATS2, and anti-YAP antibodies (D). Histogram showing average relative protein levels normalized to GAPDH (^*^ *P*<0.05).

The Hippo pathway is also regulated by cell-to-cell contact [18]. Under cell contact conditions, the Hippo pathway is activated and YAP is inactivated to prevent overgrowth and proliferation. In our study, cell clusters and cellular areas of high density were found in 50-week NP tissue. To explore whether NPC density altered YAP distribution regulation, cells were plated at three densities (Figure 7A), and YAP status was examined. In low-density cultures, YAP was primarily localized in the nucleus to promote target gene transcription and cell growth. However, in high-density cultures, cells became smaller and were in contact with each other, and YAP was primarily sequestered in the cytoplasm and restrained responding to growth inhibition (Figure 7B–7E).

### Identification of senescent NPCs

According to our *in vivo* experiments, activation of Hippo signaling resulted in decreases in YAP and CTGF gene expression in the later stage of disc injury (12 weeks post-operatively), which led to the loss of the ability of discs to self-repair. To investigate whether Hippo signaling in senescent NPCs was consistent with that *in vivo*, we induced senescent NPCs and studied changes in Hippo signaling.

### Changes in shape

**Figure 8:**
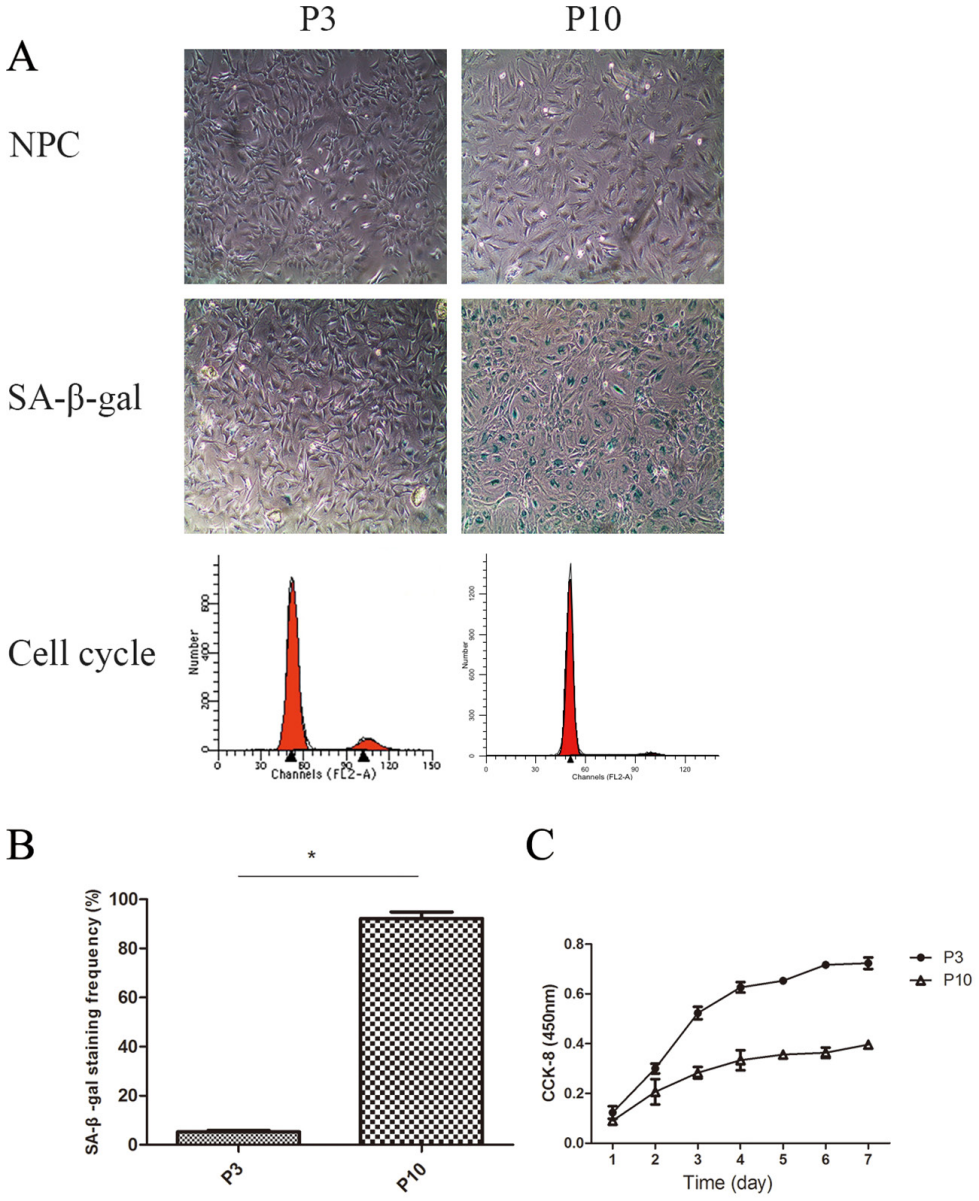
The cell proliferation potency of senescent NPCs was significantly decreased compared with that of non-senescent cells **(A)** Identification of senescent NPCs was done by morphological observation, measurement of SA-β-Gal activity, cell cycle analysis, and cholecystokinin (CCK)-8 assay. **(B)** Quantification of the percentage of positive SA-β-Gal-stained cells. **(C)** NPC proliferation in the P3 and P10 groups was analyzed using a CCK-8 assay (^*^ *P*<0.05).

When senescence is established, NPCs undergo widespread changes and develop specific characteristics. As shown in Figure 8A, NPCs and the nucleus increased in size, and cells lost their long spindle shape.

### Higher SA-β-Gal activity

SA-β-gal staining is another important characteristic of senescent cells. The mean positive percentage of SA-β-Gal staining was significantly elevated in the P10 group compared with the P3 group (Figure 8A and 8B).

### Higher percentage of G1 phase

As G1 cell cycle arrest is a marker of senescent cells, we analyzed the cell cycle of NPCs. As shown in Figure 8A, P10 group cells were easier to arrest in the G1 phase than P3 group cells.

### Lower proliferation of senescent NPCs

Senescent cells often have limited cell proliferation potency. As shown in Figure 8C, NPC proliferation in the P3 and P10 groups was analyzed using a CCK-8 assay. The cell proliferation potency of P10 cells was significantly decreased compared with that of P3 group cells.

### Hippo pathway activation in senescent NPCs

**Figure 9:**
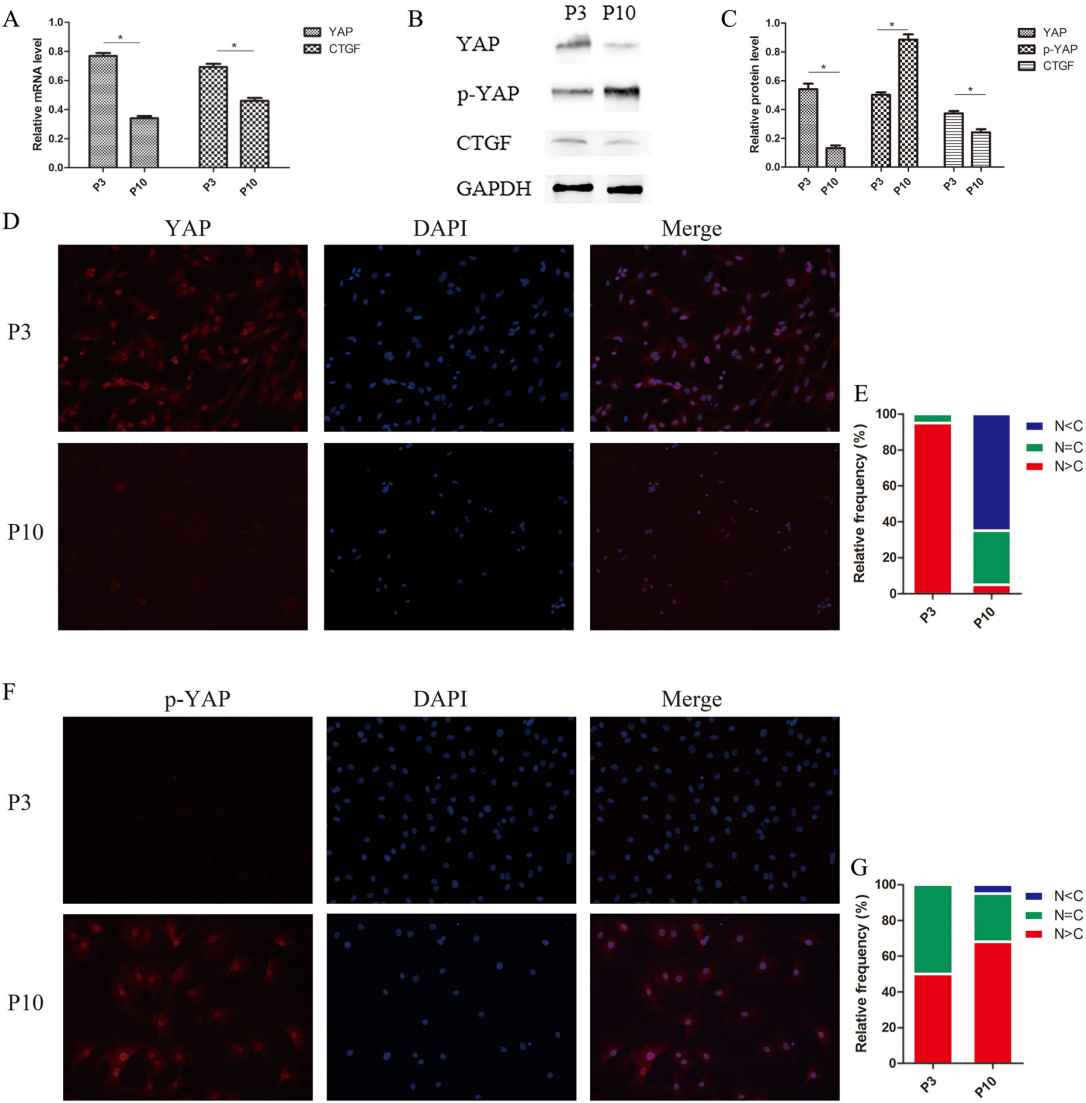
Hippo signaling activated and phosphorylated YAP, which translocated from the nucleus to the cytoplasm in senescent NPCs **(A)** The relative expression of YAP and CTGF in the P3 and P10 groups as quantified by qPCR. **(B** and **C)** Western blot analysis of Hippo pathway-related proteins using anti-YAP, anti-p-YAP, and anti-CTGF antibodies (B). Histogram showing average relative protein levels normalized to GAPDH (C). **(D** and **E)** Subcellular localization of YAP is affected by cellular status. YAP was identified with immunofluorescence staining using an anti-YAP antibody (D) and cells in five random views were quantified for YAP subcellular localization. **(F** and **G)** Subcellular localization of phosphorylated YAP is affected by cellular status. Phosphorylated YAP was identified with immunofluorescence staining using an anti-p-YAP antibody (F) and cells in five random views were quantified for phosphorylated YAP subcellular localization (G) (^*^ *P*<0.05).

In our study, Hippo signaling was strongly activated, resulting in increased YAP phosphorylation and decreased CTGF expression (Figure 9A–9C). In the P3 group, the expression of YAP increased and YAP was mainly located in the nucleus. In contrast, the senescent P10 group showed significantly decreased YAP levels in the nucleus (Figure 9D–9E). These findings suggest that NPC senescence is accompanied by activation of Hippo signaling, which inhibits cell proliferation and promotes apoptosis.

Increases in p-YAP levels suggest inhibition of cell metabolism and proliferation. Compared with group P3 cells, cell senescence in the P10 group resulted in higher p-YAP expression levels. While p-YAP was mainly localized in the nucleus in the P3 group, p-YAP levels were increased in the nucleus, as well as in the cytoplasm in the P10 group, indicating that p-YAP translocated from the nucleus to the cytoplasm in senescent NPCs (Figure 9F and 9G).

### Inhibition of YAP by lentivirus shYAP significantly induced senescence in NPCs

**Figure 10:**
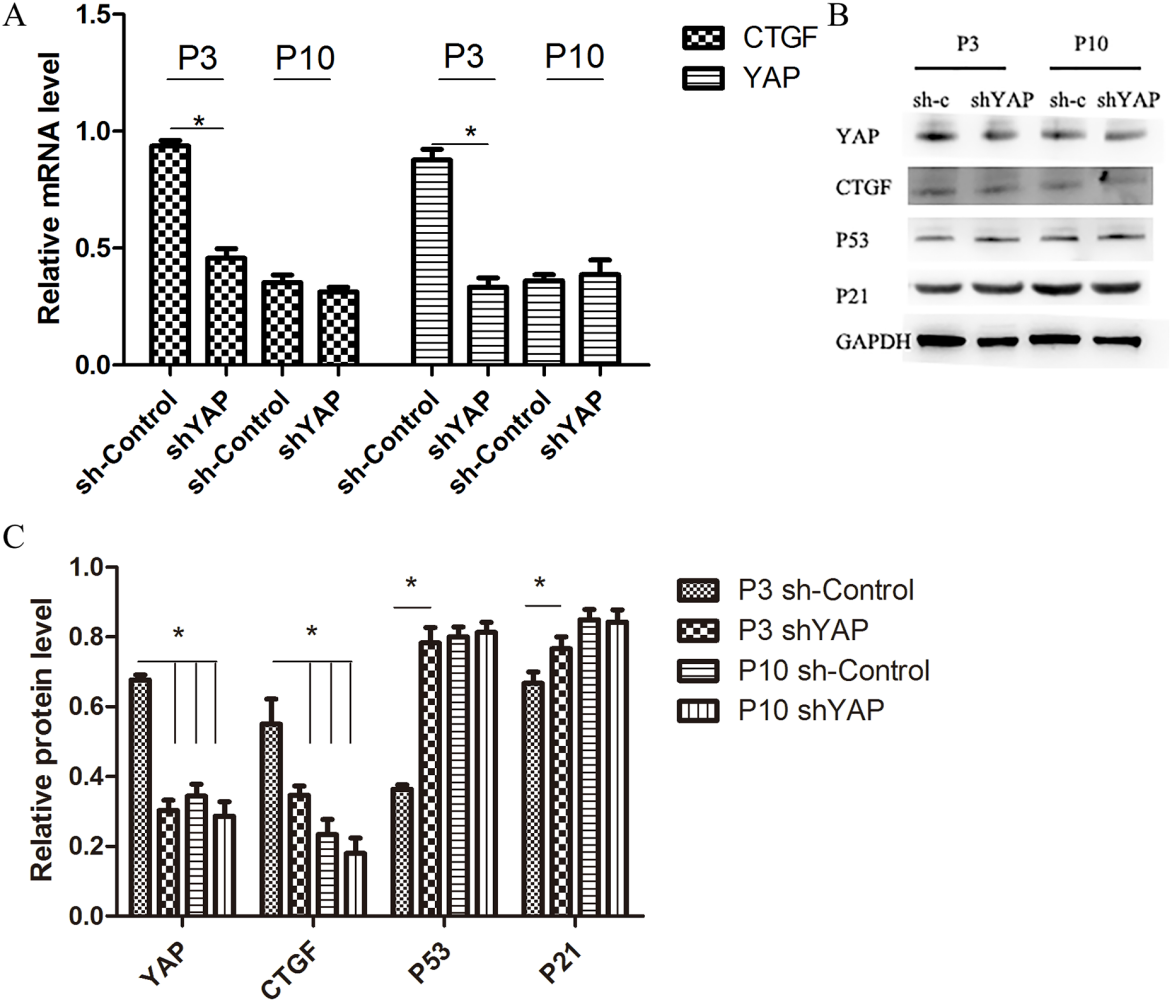
Inhibition of YAP by lentivirus shYAP significantly decreased YAP and target gene expression and induced senescence in NPCs **(A)** qPCR comparing the relative expression of YAP and CTGF in the P3 sh-Control, P3 shYAP, P10 sh-Control and P10 shYAP groups. **(B** and **C)** Western blot analysis of Hippo pathway and senescence related proteins in the P3 sh-Control, P3 shYAP, P10 sh-Control and P10 shYAP groups. groups using anti-YAP, anti-CTGF, anti-P53 and anti-P21 antibodies (B). Histogram showing average relative protein levels normalized to GAPDH (C) (^*^ *P*<0.05).

**Figure 11:**
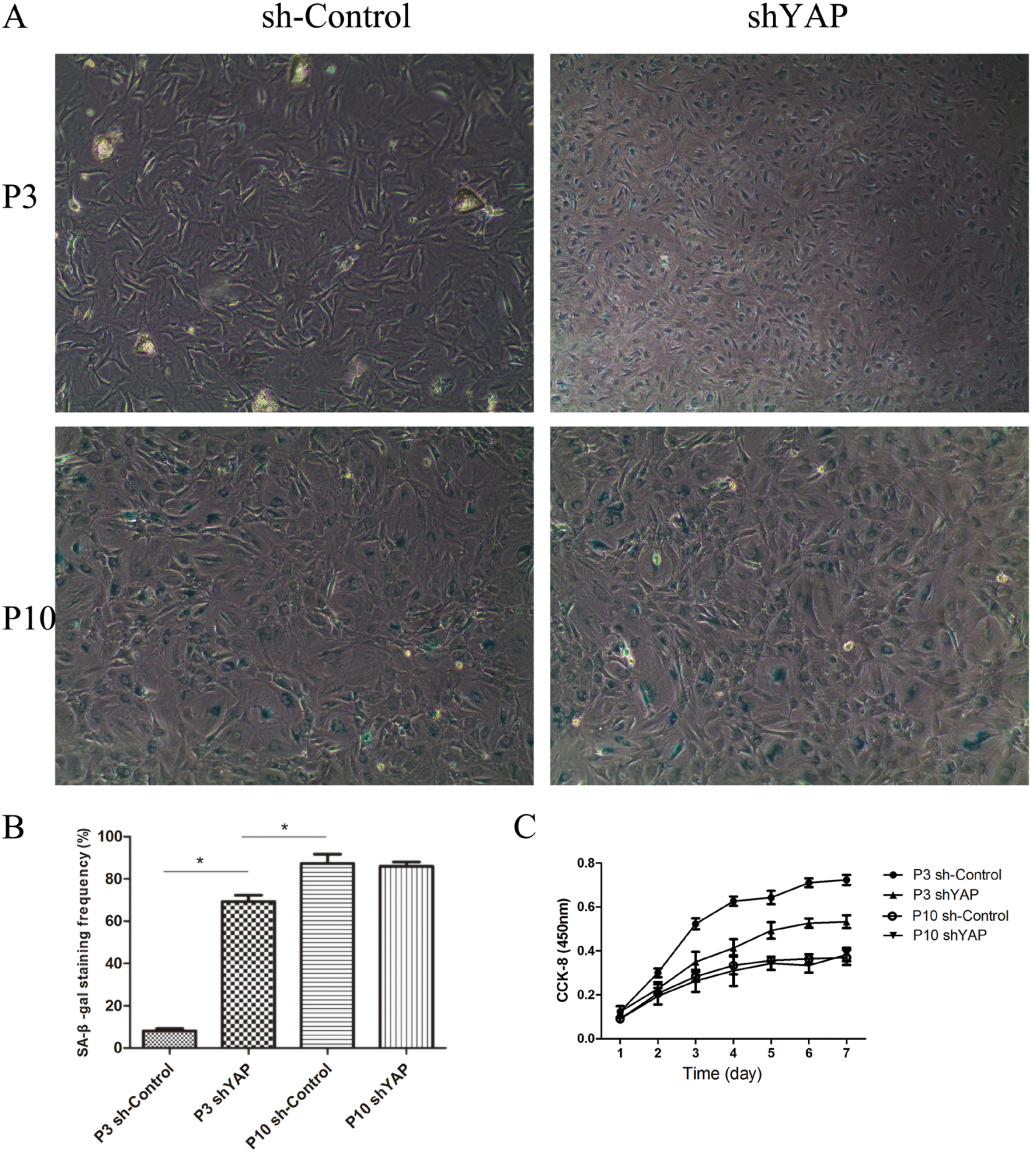
Inhibition of YAP by lentivirus shYAP significantly induced senescence in NPCs **(A)** Senescent NPCs was done by SA-β-Gal activity. **(B)** Quantification of the percentage of positive SA-β-Gal-stained cells. **(C)** NPC proliferation in the P3 sh-Control, P3 shYAP, P10 sh-Control and P10 shYAP groups was analyzed using a CCK-8 assay (^*^ *P*<0.05).

To further investigate the role of YAP regulating cell proliferation and viability in NPCs, cells were transfected with shYAP and sh-Control. The inhibitory effect of virus on YAP activation is shown in Figure 10. Compared with the P3 sh-Control group, the shYAP group markedly accelerates the premature senescence of NPCs by activating level of p53 and p21 which mediate cell replicative senescence (Figure 10A–10C) [19, 20], and this result was further confirmed by SA-β-gal staining and CCK-8 assay (Figure 11A–11C). These results suggested that the senescence of NPCs induced by interfered Hippo pathway through YAP.

## DISCUSSION

The Hippo pathway plays a vital role in maintaining tissue and cell homeostasis by regulating cell apoptosis, differentiation, and proliferation [7]. Dysregulation of Hippo signaling is associated with various human diseases [18]. However, changes that occur in the Hippo signaling pathway in IDD are unknown. In IDD, the rate of apoptosis of NPCs increases, while their proliferation is inhibited.

In the current study, the annulus puncture model was used to determine the role of the Hippo signaling pathway in regulating IDD compared with natural and injury-induced IDD. Needle puncture has become a popular animal model that mimics human IDD in rabbits, rodents, pigs, and sheep [21–24]. Although higher species have cellular components that are more similar to those of humans, their use is limited by reduced availability and higher cost. The advantages of rat tail needle puncture are that it is inexpensive and simple, with high reproducibility, thereby avoiding invasive surgical procedures in a lumbar model, which requires abdominal incision and exposure of the peritoneal cavity [25]. In this study, tail discs were punctured using a 21 G needle because use of larger needles has previously been demonstrated to result in more severe degeneration changes in DHI, Pfirrmann grades, NP loss, and reduced biomechanical stability [4]. Compared with preoperative rats, P4w and P12w rats showed lower DHI, higher Pfirrmann grades, and more severe NP loss. However, there were no significant differences in the X-ray and MRI results between these two groups, which is inconsistent with the observed changes in collagen type 2 and YAP. These results may be explained by several factors. First, annular puncture resulted in decreased DHI and loss of NP water content, as seen on MRI, which would be associated with disorganized annular lamellae and decreased nuclear pressure. Next, imaging allows only a gross observation of total disc changes and is limited in terms of observation at the protein and molecular levels. Lastly, the Pfirrmann grading system is subjective and inadequate for evaluating severe disc degeneration [26].

Nucleus tissue YAP levels decreased gradually with time, suggesting that they are closely related to the development of IDD and indicating the relationship between IDD and Hippo pathway is a dynamic balance. At the same time, increased YAP levels in acute IDD indicate that the endogenous repair route in the intervertebral disc is initiated, but the downstream target gene CTGF does not increase at the protein level. Hippo signaling may be involved in endogenous disc repair, but does not affect endogenous disc progenitor cells and stem cell niches [27].

In our study, YAP activation was accompanied by upregulation of LATS1 in young rats (4w group), resulting in a YAP-LATS negative feedback loop. The Hippo signaling pathway has been shown to be involved in tissue regeneration after damage. However, hyperactivation of YAP may cause cell overgrowth and tumorigenesis. Controlling YAP activity is important to ensure homeostasis of the cellular microenvironment and proper cell physiological regulation. Consistent with our findings, Moroishi et al. [28] previously reported that YAP activation stimulates the LATS1/2 kinases, which are negative regulators, to restrain YAP activity. When complexed with TEAD, YAP stimulates LATS2 transcription and indirectly activates LATS1/2 kinase through angiomotin (AMOT) accumulation and neurofibromatin 2 (NF2) induction.

**Figure 12:**
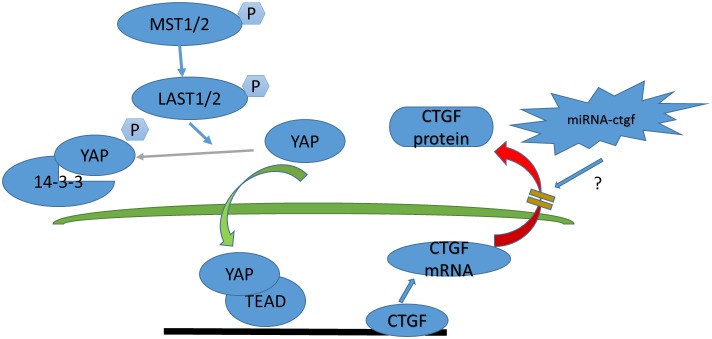
Model depicting Hippo signaling pathway regulation in acute disc injury-induced IDD

**Figure 13:**
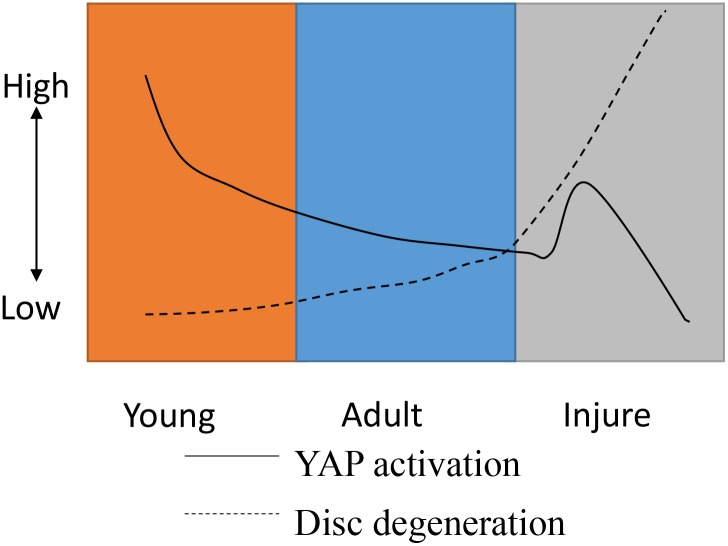
A model for YAP regulation and disc degeneration caused by natural development and injury

To determine how Hippo signaling changes during chronic IDD, we performed H&E staining, immunohistochemistry, western blotting, and qPCR on tissues from 12-week postoperative rats. Surprisingly, the Hippo pathway was strongly activated and YAP phosphorylation was increased. These effects may be due the endogenous repair potential of acute IDD, which low self-repair ability is intrinsic nature [29, 30]. Moreover, a CTGF post-transcriptional regulation mechanism, such as micro-ctfg, may be involved (Figure 12). YAP was not observed in the intervertebral discs of the P12w group in this study. Nevertheless, in the natural degeneration group, YAP levels in the NP and CE decreased gradually with age (Figure 13).

Intervertebral discs are tissues that lack blood vessels. Thus, NPCs rely on convection and diffusion for nutrient and metabolite exchange, principally with capillaries in the adjacent vertebral bodies [31, 32]. Nachemson previously demonstrated that cartilage endplate capillaries are more numerous at the disc center [33], which is the principal route for nuclear nutrition [34]. Nutrition transport is critical to disc health, as cells regulate the biochemical environment and maintain the extracellular matrix [35]. It is suggested that the CE may play an important role in the course of IDD. Further investigation of the biological mechanism of Hippo signaling in CE is warranted.

As shown by H&E staining, the disappearance of notochordal cells and formation of cell clusters in the P50w and P4w groups were correlated with IDD degeneration. Decreased proteoglycan synthesis is associated with the loss of notochordal cells, resulting in reduced mechanical function. Moreover, different cell density growth conditions have differential effects on Hippo signaling *in vivo*. A high density of NPCs acts similarly to the presence of cell clusters; thus, in IDD, a large number of chondrocyte-like NPCs are formed in the degenerated intervertebral disc. As a result, YAP is activated at low cell density, whereas YAP is inactive at high cell density, resulting in the translocation of YAP from the nucleus to the cytoplasm, where it cannot exert its effects. Previous studies have shown that at high cell density, YAP exits the nucleus, indicating growth inhibition [36, 37]. In addition, we observed decreased total YAP levels in NPCs seeded at high densities compared with NPCs seeded at low densities. Cell-cell contact at high density produces a growth inhibitory signal that is in large part mediated by the Hippo pathway [38, 39].

To investigate the regulation of Hippo signaling in senescent NPCs, and to compare it with chronic disc degeneration *in vivo*, the replication senescence method was used to induce senescence of NPCs. Senescent NPCs have low growth and high apoptosis rates. In the present study, Hippo signaling was activated in senescent NPCs, producing a growth inhibitory signal. In senescent cells, YAP was mainly localized in the cytoplasm, whereas in non-senescent cells, YAP levels in the nucleus increased. Interestingly, p-YAP was mainly concentrated in the nucleus, but as cell senescence progressed, p-YAP levels in the nucleus decreased and those in the cytoplasm increased.

To reveal the relationship of Hippo pathway and senescence, a shYAP was applied in our study. We found that the beneficial effects were lost when the activation of YAP was inhibited, indicating that the Hippo pathway modulate NPCs proliferation and senescence via regulation of YAP. In addition, our study also shown the limited role of YAP in over-senescence cells and the molecular mechanisms still need further investigation, such as senescence cell low proliferation and limited cell division ability.

In summary, our study demonstrates that the relationship between the Hippo pathway and the dysregulation of YAP may play a pivotal role in natural and injury-induced IDD progression. The expression of the CTGF gene was not consistent with protein, considering the incomplete compensation of self-repair. Meanwhile, the Hippo pathway was also shown to be regulated by cell density and cell senescence. These finding demonstrate the complexity of cell fate determination mechanisms, and only up-regulation of YAP may not contribute to NPC proliferation and anti-degeneration in intervertebral discs.

## MATERIALS AND METHODS

### Annulus fibrous needle puncture

Rats (10 weeks) were anesthetized using an intraperitoneal injection of 10% chloral hydrate (0.3 ml/100 g). They were placed in the prone position and the tail skin was disinfected with ethanol. The coccygeal 5-6, 6-7, 7-8, and 8-9 discs were identified and percutaneously punctured using a 21 G needle with a stopper to a depth of 5 mm, whereupon the needle was rotated 360° and held for 30s.

Animals were maintained under a 12-h light/dark cycle and allowed free activity in their cages. Analysis was performed at 4 weeks (P4w) and 12 weeks (P12w) post-operatively. A natural IDD group and a control group (healthy rats with no lesions) were also included and analyzed at 4 weeks (4w), 14 weeks (14w) as P4w control, 22 weeks (22w) as P12w control and 50 weeks (50w). All experiments were reviewed and approved by the Institutional Animal Care and Use Committee in Southeast University School of Medicine, and were conducted according to the committees’ guidelines.

### Calculation of disc height index

Rats were placed in the prone position and their tails were laid straight on a platform. Vertical X-rays were taken, focusing on the target levers. The disc height index (DHI) was calculated from the digital radiographs using ImageJ software. The %DHI was calculated as the post-operative DHI divided by the pre-operative DHI, as described by Han et al [40].

### MRI examination and Pfirrmann grade evaluation

Immediately after X-ray examination, the rat tails were scanned using 7.0 T magnetic resonance imaging (MRI). The midsagittal section of the tail was identified and examined for disc degeneration using Pfirrmann grading [26].

### Hematoxylin and eosin staining

All animals were sacrificed using CO_2_ inhalation and the coccygeal 5-6, 6-7, 7-8, and 8-9 vertebral segments were dissected. The specimens were fixed in 10% formalin for 20 h at room temperature, decalcified in an ethylenediaminetetraacetic acid (EDTA)-glycerol solution for 4 weeks, paraffin-embedded, sectioned sagittally to 5-μm thickness, and stained with hematoxylin and eosin (H&E).

### Immunohistochemical detection of collagen type 2 and YAP

The sections were treated with proteinase K and blocked in 3% bovine serum albumin (BSA). Sections were incubated at room temperature for 1 h with primary antibody. A horseradish peroxidase (HRP)-labeled secondary antibody was added to the specimens and incubated for 30 min at 37°C in a black humidity chamber.

### Quantitative polymerase chain reaction

Total RNA was isolated from nucleus pulposus (NP) tissue or NPCs using TRIzol reagent (Invitrogen). cDNA was synthesized by reverse transcription using random hexamers and subjected to quantitative polymerase chain reaction (qPCR) with gene-specific primers (Table 1) in the presence of SYBR Green One. The relative abundance of mRNA was calculated using the 2^-ΔΔct^ method.

**Table 1:** Sequences of primers for qPCR

Gene		Primers
LATS1	Forward	ACCAGAAGACCGTCTAGGCA
	Reverse	TTCCTCGTTACCATCGCTCC
LATS2	Forward	ACCATGCTGCTGTTACTCCC
	Reverse	CGGTCTTCAGGGCTTCCTTT
YAP	Forward	ATTTCGGCAGGCAATACGGA
	Reverse	AGCTAATTCCCGCTCTGACG
CTGF	Forward	GGCGTAAAGCCAGGGAGTAA
	Reverse	CTCACTTCGGTGGGGTGTTT
GAPDH	Forward	ACAGCAACAGGGTGGTGGAC
	Reverse	TTTGAGGGTACAGCGAACTT

### Western blot analysis

NP tissue or NPCs were lysed in radioimmunoprecipitation assay (RIPA) buffer. The proteins were separated on sodium dodecyl sulfate (SDS)-polyacrylamide gels and transferred onto nitrocellulose membranes. The blots were detected with antibodies against LATS1, LATS2, YAP, p-YAP, and CTGF and normalized to the loading control glyceraldehyde 3-phosphate dehydrogenase (GAPDH) (Table 2). Signals were detected and quantified using ImageJ software.

**Table 2:** Primary antibodies

Antibody	Company (catalog number)
LATS1	Abcam (ab70562)
LATS2	Antibodies-online (ABIN753568)
YAP	Santa Cruz (sc-376830)
Phosphorylated YAP (Ser 127)	Abcam (ab172374)
CTGF	Abcam (ab6992)
p53	Cell Signaling Technolog (32532)
p21	Bioss (bs-0741P)
GAPDH	Bioss (bs-2188R)

### Immunofluorescence staining

NPCs were cultured on slides to the appropriate density and fixed with 4% paraformaldehyde for 15 min and then permeabilized with 0.1% Triton X-100. After blocking with 5% BSA for 30 min, slides were incubated with primary antibodies against YAP and phosphorylated YAP diluted in 5% BSA for 2 h. The slides were washed with phosphate-buffered saline (PBS) three times and incubated with the Alexa Fluor 488-conjugated secondary antibody (1:1,000 dilution) for 2 h. The slides were then washed and mounted.

### NPC culture and senescent cell induction and identification

NPCs were cultured in Dulbecco's modified Eagle's medium (DMEM)/F12 with 10% fetal bovine serum (FBS) and 1% penicillin/streptomycin. Cells from the third passage were plated at a low density (3 × 10^5^), medium density (1 × 10^6^), and high density (2 × 10^6^) per well into 6-well plates. Twelve hours after plating, the medium was replaced with serum-free DMEM/F12 and cultured for 8 h. Because *in vitro* subcultivation (replication senescence) can lead to senescence of NPCs [41], the control passage 3 (P3) cells were used to induce the premature senescence of cells (P10) according to our preliminary work. The cells were plated at a density of 1 × 10^6^ cells per well into 6-well plates. A cholecystokinin (CCK)-8 assay and cell cycle analysis were performed and SA-β-Gal activity was measured according to the manufacturer's instructions.

### Virus transfection

Lentivirus packaging cells were transfected with GV248-hU6-MCS-Ubiquitin-EGFP-IRES-puromycin vector (GeneChem, Shanghai, China) containing either the YAP knockdown (shYAP) or a negative control sequence (sh-Control). Sequences of the shYAP were as follows: sense strand 5’-ccgggaTCCCTGATGATGTACCA TTctcgagAATGGTACATCATCAGGGAtctttttg-3’, and sequences of the sh-Control were as follows: sense strand 5’-TTCTCCGAACGTGTCACGT-3’. P3 and P10 groups cells were seeded in a six-well plate 24 h at medium density (1×106) before transfection. After 24 h, cells were transfected with shYAP or sh-Control duplexes for 36 h according to the manufacturer's instructions. After the following further treatments, cells were harvested for western blot, qPCR, SA-β-gal staining and CCK-8 assay.

### Statistical analysis

All results were expressed as the mean±SD of data obtained in triplicate, from at least three individual experiments. All statistical analyses were performed with Excel software (Microsoft, Bellevue, WA), and GraphPad Prism Version 6.0 software (GraphPad Software, La Jolla, CA). *P* values <0.05 were considered statistically signifcant.

The English in this document has been checked by at least two professional editors, both native speakers of English. For a certificate, please see: http://www.textcheck.com/certificate/eKCCx6

## SUPPLEMENTARY MATERIALS FIGURES AND TABLES



## Abbreviations

YAP: yes-associated protein
LATS: large tumor suppressor kinases
