# Supplementary material for: Dysregulation of YAP by the Hippo pathway is involved in intervertebral disc degeneration, cell contact inhibition, and cell senescence
Source: Oncotarget. 2017 Dec 14;9(2):2175–92. doi: 10.18632/oncotarget.23299 (PMC5788631; doi:10.18632/oncotarget.23299)
Supplement: Supplementary file 1 [file oncotarget-09-2175-s001.pdf]

Dysregulation of YAP by the Hippo pathway is involved in intervertebral disc degeneration, cell contact inhibition, and cell senescence

SUPPLEMENTARY MATERIALS

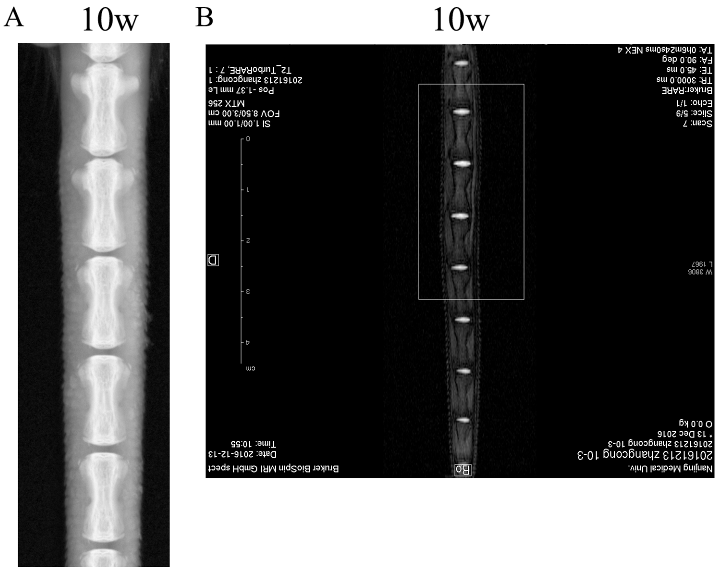

Supplemental Figure 1: Rat tail X-ray **(A)** and magnetic resonance imaging **(B)** examination at 10 weeks.
